# Omega-3 polyunsaturated fatty acids status and cognitive function in young women

**DOI:** 10.1186/s12944-019-1143-z

**Published:** 2019-11-06

**Authors:** Rebecca L. Cook, Helen M. Parker, Cheyne E. Donges, Nicholas J. O’Dwyer, Hoi Lun Cheng, Katharine S. Steinbeck, Eka P. Cox, Janet L. Franklin, Manohar L. Garg, Helen T. O’Connor

**Affiliations:** 1 Faculty of Health Sciences, Discipline of Exercise and Sport Science, The University of Sydney, PO Box 170, Lidcombe, NSW 1825 Australia; 2 Charles Perkins Centre, The University of Sydney, Camperdown, NSW Australia; 3 School of Exercise Science, Sport and Health, Charles Sturt University, Bathurst, NSW Australia; 4 Academic Department of Adolescent Medicine, The Children’s Hospital at Westmead, Westmead, NSW Australia; 5 Faculty of Medicine and Health, Sydney Medical School, Discipline of Child and Adolescent Health, The University of Sydney, Westmead, NSW Australia; 6 Metabolism and Obesity Services, Royal Prince Alfred Hospital, Camperdown, NSW Australia; 7 School of Biomedical Sciences and Pharmacy, University of Newcastle, Callaghan, NSW Australia

**Keywords:** Omega-3, N-3 PUFA, Cognition, Attention, Women

## Abstract

**Background:**

Research indicates that low omega-3 polyunsaturated fatty acid (n-3 PUFA) may be associated with decreased cognitive function. This study examined the association between n-3 PUFA status and cognitive function in young Australian women.

**Methods:**

This was a secondary outcome analysis of a cross-sectional study that recruited 300 healthy women (18–35 y) of normal weight (NW: BMI 18.5–24.9 kg/m^2^) or obese weight (OB: BMI ≥30.0 kg/m^2^). Participants completed a computer-based cognition testing battery (IntegNeuro™) evaluating the domains of impulsivity, attention, information processing, memory and executive function. The Omega-3 Index (O3I) was used to determine n-3 PUFA status (percentage of EPA (20:5n-3) plus DHA (22:6n3) in the red cell membrane) and the participants were divided into O3I tertile groups: T1 < 5.47%, T2 = 5.47–6.75%, T3 > 6.75%. Potential confounding factors of BMI, inflammatory status (C-reactive Protein), physical activity (total MET-min/wk), alpha1-acid glycoprotein, serum ferritin and hemoglobin, were assessed. Data reported as *z*-scores (mean ± SD), analyses via ANOVA and ANCOVA.

**Results:**

Two hundred ninety-nine women (26.9 ± 5.4 y) completed the study (O3I data, *n* = 288). The ANOVA showed no overall group differences but a significant group × cognition domain interaction (*p* < 0.01). Post hoc tests showed that participants in the low O3I tertile group scored significantly lower on attention than the middle group (*p* = 0.01; ES = 0.45 [0.15–0.74]), while the difference with the high group was borderline significant (*p* = 0.052; ES = 0.38 [0.09–0.68]). After confounder adjustments, the low group had lower attention scores than both the middle (*p* = 0.01) and high (*p* = 0.048) groups. These findings were supported by univariate analyses which found significant group differences for the attention domain only (*p* = 0.004).

**Conclusions:**

Cognitive function in the attention domain was lower in women with lower O3I, but still within normal range. This reduced but normal level of cognition potentially provides a lower baseline from which cognition would decline with age. Further investigation of individuals with low n-3 PUFA status is warranted.

## Background/objectives

Research on n-3 PUFA includes its potential role in optimizing brain health and cognitive function [1–5]. Evidence also indicates that maintaining adequate body levels of n-3 PUFA is important for optimizing health across the lifespan [1–5]. While prevalence rates for low n-3 PUFA status is lacking in Australia, studies in Australian women highlight suboptimal intakes of n-3 PUFA, [6, 7] and results from the Australian Health Survey [8] indicate young women are not meeting the suggested dietary target (SDT) for chronic disease prevention, [9] with mean intake falling at 41% of the SDT [8]. Although beneficial associations between n-3 PUFA status and cognitive performance have been reported, the evidence is equivocal, with systematic reviews and meta-analyses indicating no significant effect of n-3 PUFA supplementation on cognitive performance in adults [2–5, 10].

One factor that may reduce the capacity to observe significant effects or associations is the use of plasma n-3 PUFA assessment. The Omega-3 Index (O3I) is a measure of erythrocyte eicosapentaenoic acid (EPA) and docosahexaenoic acid (DHA) and is known to be a more accurate measure of n-3 PUFA status than serum or plasma because it is indicative of omega-3 exposure over the previous 3 months (reflecting the life cycle of erythrocytes), rather than recent dietary intake [11]. Another factor is the influence of confounding variables on cognitive function. Obesity and its associated co-morbidities, including metabolic disease and systemic inflammation, are particularly relevant as there is increasing evidence for their association with cognitive decline [12–19]. Physical inactivity, which is often strongly associated with obesity, has also been linked with cognitive decline or reduced cognitive performance [20–24]. Iron status also may impact cognitive function [25, 26]. The impact of these confounding variables should be considered when examining the relationships between n-3 PUFA status and cognitive function, but unfortunately, studies examining these relationships rarely measure or adjust for these.

Given evidence of sub-optimal n-3 PUFA intake reported in Australian women, [6, 7] the aim of this study was to examine in young women the relationship between cognitive function and n-3 PUFA status measured by the O3I. As obesity and physical inactivity currently are increasing in this population group, [27–29] the study also examined the potential confounding effects of these factors (and additionally, of obesity-related systemic inflammation) on cognitive function. The impact of iron status was also examined because of recent evidence for its influence on attention [25].

## Subjects/methods

### Study design and participants

As reported elsewhere (Cook et al., 2017), [25] this cross-sectional study (Food, Mood & Mind) was designed with the primary aim of assessing the iron status and cognitive function in young (18–35 y) normal weight (NW) (body mass index (BMI): 18.5–24.9 kg/m^2^) and obese weight (OB) (BMI: ≥30.0 kg/m^2^) women. A secondary aim was to assess the association between n-3 PUFA status (measured by O3I) and cognitive function and this secondary outcome analysis is reported here. Obesity, physical activity levels, systemic inflammation, alpha1-acid glycoprotein and iron status were evaluated as potential confounding factors. Participants were recruited from both metropolitan (Sydney) and rural (Bathurst) areas of Australia. Sample size was calculated with respect to the primary outcome (iron and cognition) and the recruitment target was 300 healthy women [25]. The study was approved by the associated Human Research Ethics Committees of the participating universities and local health district services (protocol numbers X10–0259, HREC/10/RPAH/455 and 2014/050).

### Inclusion and exclusion criteria

Participants were eligible if they were healthy and had a BMI in the normal weight or obese category according to the WHO guidelines (BMI 18.5–24.9 and ≥ 30.0 kg/m^2^ respectively) [30]. Exclusions were significant medical conditions; disorders or medications which may compromise cognitive function, vision, hearing, or motor coordination; smoking; alcohol consumption ≥50 g per week; and current pregnancy or breastfeeding. Participants were required to be literate in English.

### Data collection

Participants attended two separate appointments (at a university laboratory or an obesity clinic within a major university teaching hospital) approximately 1 week apart. The first session involved informed consent, anthropometric measurements (weight, height, waist circumference), cognitive testing and a battery of questionnaires. All testing was conducted after breakfast and prior to 1300 h. Participants were asked to refrain from heavy exercise, alcohol and caffeine (and other stimulants) on the morning of the cognition test and to consume their usual breakfast to ensure that they were not hungry during the testing session. Caffeine was excluded as it has been shown to reduce fatigue and help increase attention. The second visit involved collection of a fasting blood sample, thus avoiding the fasting state for cognitive testing.

### Cognition assessment

Cognitive function was assessed using a validated touch-screen cognition testing platform, IntegNeuro™ (©Brain Resource Company) [31]. Performance on a total of 14 tests is used to formulate *z*-scores across five cognitive domains: attention, impulsivity, information processing, memory and executive function. *Z*-score normal range is + 1 to − 1 standard deviation and scores are adjusted for age, sex and years of education. Positive scores reflect better than average performance while negative scores reflect lower than average performance. Outliers lower than − 4 *z*-score were excluded from the analysis (< 4%, *n* = 11); there were no outliers greater than + 4 *z*-score.

### Blood collection and biochemical analysis

Biochemical analysis was performed by a National Association of Testing Authorities (NATA) Australia accredited diagnostic laboratory. Inflammation was analyzed via C-reactive Protein (CRP) using CRP Rate Nephelometry on COBAS 8000 e602 and alpha-1 acid glycoprotein (α1GP) using immunoturbidimetry on a Konelab 20XT Clinical Chemistry Analyzer (using reagent from Thermo Fisher), with CRP > 5.0 mg/L and/or α1GP > 1.0 g/L indicative of raised levels of inflammation (CRP reference range ≤ 5.0; α1GP reference range ≤ 1.0) [32, 33]. Hemoglobin (Hb) was measured via absorption spectrophotometry and flow cytometry on the Abbot CELL-DYN Sapphire System (Hb reference range 120-150 g/L). Iron markers were analyzed via automated immunoassay on the Roche COBAS 8000 e602 and the raw serum ferritin (SF) values (reference range 20-300μg/L) were corrected for inflammation as described previously [25].

The O3I analysis was conducted at a university laboratory using trans-esterification of the washed erythrocyte fraction of blood followed by gas chromatography using a fixed carbon-silica column 30 m × 0.25 mm (DB-225) (J and W Scientific) [34]. The O3I was used as a reliable indicator of overall n-3 PUFA status and was calculated by addition of the percentage of EPA (20:5n-3) and DHA (22:6n-3) in each erythrocyte sample as proportions of total erythrocyte membrane fatty acids in each sample [11, 33–35].

### Physical activity assessment

The short form of the International Physical Activity Questionnaire (IPAQ) was used to assess overall level of physical activity (PA) in Metabolic Equivalent of the Task (MET)-minutes (min)/week (wk). The IPAQ allows collation of comparable estimates of PA and has been validated transnationally [36, 37].

### Statistical analyses

In order to explore the relationship between n-3 PUFA status measured by the O3I and cognitive function, the participants were divided into O3I tertile groups (*n* = 96 per group). Initial one-way analyses of variance (ANOVAs) were conducted on demographic characteristics and the potential confounders of BMI, CRP, PA, α1GP, SF and Hb. The relationship between O3I and cognitive function was then investigated using two-way (3 × 5) ANOVAs with O3I tertiles as an independent groups factor (T1, T2, T3) and the five *z*-scores (impulsivity, attention, information processing, memory, executive function) as a repeated measures factor. Tukey’s post hoc tests were used in all cases to determine the precise locus of any significant differences observed. Subsequently, the variables of BMI, CRP, PA, α1GP, SF and Hb were added as covariates to the 3 × 5 ANOVA models. Univariate analyses were also carried out within each ANOVA and ANCOVA to examine group differences on the *z*-scores for each cognitive domain. Significance for all analyses was set at *p* < 0.05. In the text and tables, all results are reported as mean ± standard deviation (SD) and between-group differences as effect size (ES) ± 95% confidence interval (CI). All statistical analyses were carried out using Statistica software (v.12; StatSoft Inc., Tulsa, OK, USA).

## Results

**Fig. 1:**
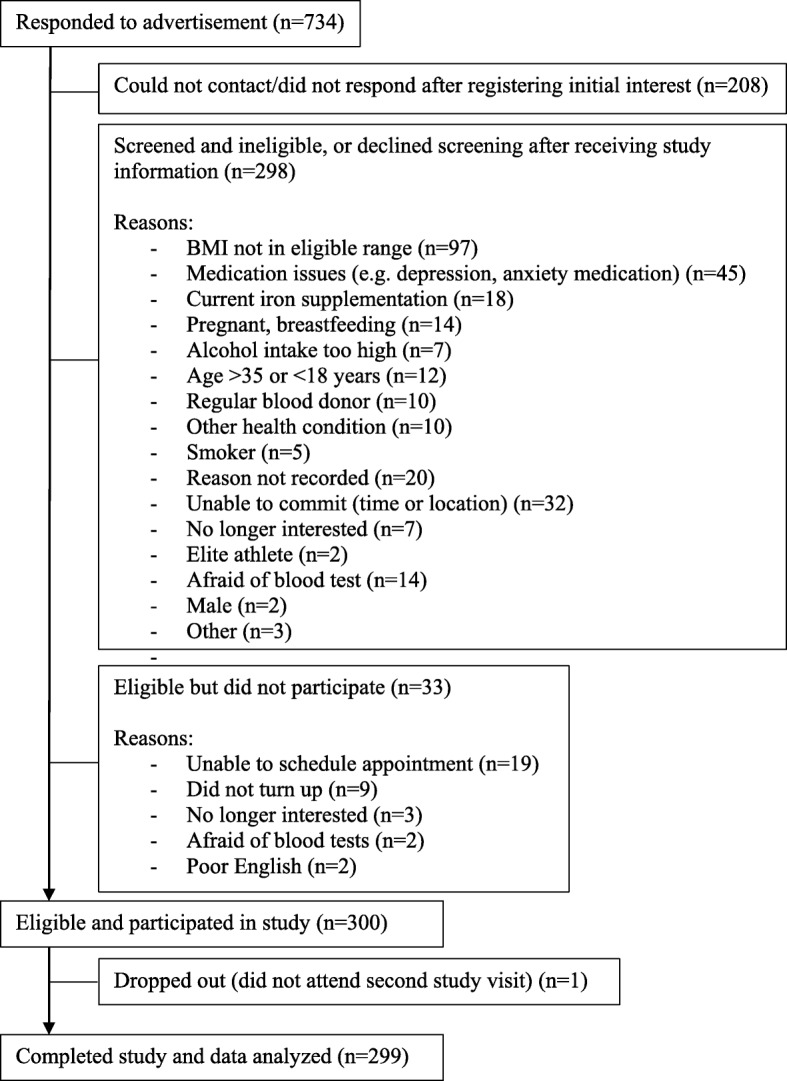
Participant recruitment and study participation flow chart

The screening and recruitment of participants are summarized in Fig. 1. Although 299 women completed the study, blood samples could not be assayed in 11 cases, hence the analysis was based on 288 participants.

The demographic characteristics of the tertile groups, as well as their biochemical markers, are shown in Table 1. Years of education varied significantly across the groups (*p* = 0.02), the post hoc tests showing that the lowest O3I tertile group had about 1 year less education than the highest group (p = 0.02) while the difference from the middle group was not significant (*p* = 0.10). Of the five covariates, the O3I groups did not differ significantly on PA, SF or Hb, but there were significant group differences in BMI (and weight), CRP and α1GP levels. Post hoc pairwise comparisons showed that the lowest O3I group differed significantly from the highest group on these three variables (*p* ≤ 0.02) but differed significantly from the middle group only on α1GP levels (*p* = 0.03). There were proportionately more participants with normal weight in the highest tertile group and more participants with obesity in the lowest tertile group (*p* < 0.0001).

**Table 1:** Demographics and biochemical markers (mean ± SD)

	Total group (*n* = 288)	Tertile 1 < 5.47	Tertile 2 = 5.47–6.75	Tertile 3 > 6.75	*P*-value^a^
		(n = 96)	(n = 96)	(n = 96)	
Omega-3 Index (%)	6.3 ± 1.7	4.7 ± 0.8	6.1 ± 0.4	8.1 ± 1.5	< 0.0001
Age (y)	25.8 ± 5.1	25.3 ± 5.2	25.7 ± 5.2	26.2 ± 4.8	0.47
Education (y)	16.2 ± 2.2	15.7 ± 2.2	16.3 ± 2.1	16.6 ± 2.2	0.02
Weight (kg)	78.0 ± 22.9	85.6 ± 24.5	80.2 ± 22.7	68.0 ± 17.7	< 0.0001
BMI (kg/m^2^)	28.5 ± 8.4	31.6 ± 9.3	28.9 ± 8.2	25.1 ± 6.0	< 0.0001
Normal weight 18.5–24.9	150	34	48	68	< 0.0001
Obese weight ≥ 30.0	138	62	47	28	
CRP (mg/L) *Normal Range: < 5 mg/L*	3.3 ± 4.2	4.0 ± 4.7	3.6 ± 4.2	2.3 ± 3.3	0.01
α1GP (mg/L) *Normal Range: < 1 g/L*	0.74 ± 0.22	0.82 ± 0.21	0.75 ± 0.21	0.66 ± 0.22	< 0.0001
Physical Activity (MET-min/wk)	2634 ± 2144	2641 ± 2121	2542 ± 2251	2718 ± 2074	0.85
SF (corrected) ug/L *Normal range 20–300 g/L*	42.9 ± 31.8	37.6 ± 26.4	44.4 ± 38.9	46.7 ± 28.2	0.12
Hb (g/L) *Normal Range: 120–150 g/L*	134.4 ± 9.8	135.9 ± 10.2	133.9 ± 10.0	133.3 ± 9.1	0.17

^a^One-way ANOVA on all variables except Chi-square test for weight category distributions. *Abbreviations*: *O3I* omega-3 index, *BMI* body mass index, *CRP* C-reactive Protein, *α1GP* alpha1-acid glycoprotein, *MET* metabolic equivalent of task, *min* minute, *wk*. week, *SF* serum ferritin, *Hb* hemoglobin. Missing data: α1GP (*n* = 10), Hb (n = 2)

**Fig. 2:**
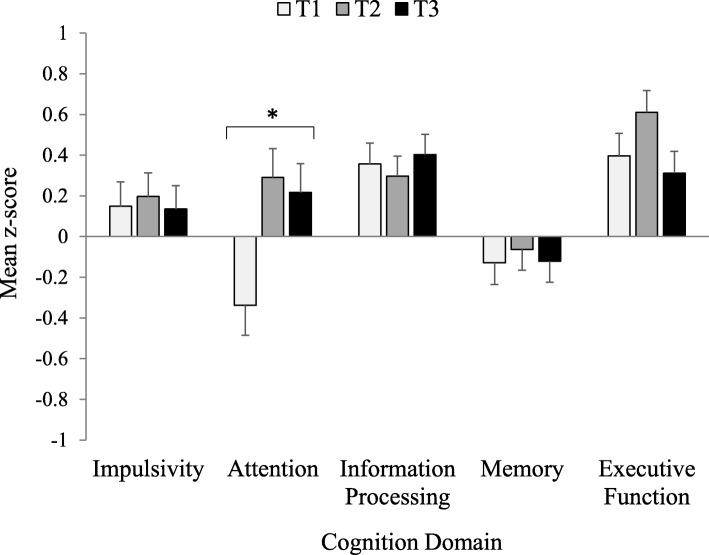
Omega-3 Index tertile groups (T1, T2, T3) versus cognitive performance in young women. Mean *z*-score ± SE (standard error); normal *z*-score is + 1 to − 1. *p = 0.004 on univariate analysis

The cognitive function of the three groups was assessed across the five domains. The mean *z*-scores for each domain across all O3I tertile groups were in the normal range (Fig. 2). The ANOVA showed no significant overall difference between the groups (*p* = 0.22) but there was a significant interaction between groups and cognitive domains (*p* < 0.01). Examination of the mean values in Fig. 2 shows that the locus of this interaction was the attention domain, with the lowest tertile group having a lower score than the other two groups. Post hoc tests on this interaction confirmed that the lowest tertile group scored significantly lower on attention than the middle group (*p* = 0.01; ES = 0.45 [0.15–0.74]), while the difference with the highest group was borderline significant (*p* = 0.052; ES = 0.38 [0.09–0.68]). The middle and highest O3I groups had similar attention scores. Univariate analyses on each domain across the three groups confirmed a significant group effect only for attention (*p* = 0.004).

Of the five covariates, only BMI and CRP showed significant (weak) correlations with cognition domains, specifically attention and memory (r = − 0.14 to − 0.22, *p* ≤ 0.02). BMI and CRP were also found to be positively correlated with each other (r = 0.67, *p* < 0.0001). Consequently, only the analyses of covariance with these two covariates had any effect relative to the unadjusted model and so only they will be reported. Similar to the unadjusted model, there were no significant overall group effects after adjusting individually for BMI (*p* = 0.28) and CRP (*p* = 0.23). The group × domain interaction was still significant for CRP (*p* = 0.013) but now borderline for BMI (*p* = 0.053), the locus again being the attention domain. Given these *p* values, post hoc tests were carried out in both cases and showed that for BMI, the lowest O3I group scored significantly lower on attention than both the middle (p = 0.01) and highest (*p* = 0.047) O3I groups, while for CRP, the lowest group scored significantly lower on attention than the middle group (p = 0.01) and was borderline significant with the highest group (*p* = 0.050). The middle and highest O3I groups again had similar performance scores. The univariate analyses on each domain across the three groups again confirmed significant effects for attention only (*p* ≤ 0.029).

When these two covariates were combined in one ANCOVA model, the pattern of results mirrored that found after adjusting for BMI alone (the lack of further effect of CRP can be attributed to the degree of collinearity between these covariates). There was no significant group effect (*p* = 0.27) and the group x domain interaction was again borderline (*p* = 0.054). Given this *p* value, post hoc pairwise comparisons were carried out and again showed that the lowest O3I group had significantly lower performance on attention than both the middle (*p* = 0.01) and highest (*p* = 0.048) O3I groups. The middle and highest O3I groups had similar attention scores. The univariate analyses were again significant for the attention domain (*p* = 0.029).

In summary, no overall group effect was observed with either the ANOVA or ANCOVA models but in each case, post hoc tests on significant group x domain interactions revealed a pattern whereby the lowest O3I tertile group scored significantly lower than the middle and highest tertile groups in the cognitive domain of attention, with the latter groups showing similar scores. These findings were supported in each case by the univariate analyses, which found significant group differences for the attention domain (Fig. 2). When these ANOVA models were repeated on quartile (*n* = 72) and quintile (*n* = 57) O3I groups, a similar pattern of results was observed. There were no overall group effects but there were significant group × domain interactions (*p* < 0.02) on which post hoc tests revealed that the lowest quartile and quintile groups scored lowest in the domain of attention. Again, significant group differences in the attention domain were observed on univariate analyses (*p* < 0.01).

## Discussion

This cross-sectional study examined the association of O3I with cognitive function in young, healthy, normal weight and obese women. While the cognitive performance of the participants was within the normal range, the study provides evidence for decreased performance in the attention domain in women with a lower Omega-3 Index. The major significant group differences remained after adjustment for known confounders, with the post hoc tests still significant. Of the five confounders, BMI appeared to be most strongly linked to cognition. While a cut-off for n-3 PUFA intake for optimal brain health in young adults cannot be suggested at this stage, the lowest O3I tertile range (< 5.47%) may be sub-optimal for cognitive function. The reduced but normal level of cognition associated with lower n-3 PUFA potentially provides a lower baseline from which cognition would continue to decline with age.

There are a relatively small number of studies examining the effects of n-3 PUFA on cognitive function in young adults, ranging from randomized controlled trials to observational studies [1, 7, 38–45]. The majority of studies include psychometric tests measuring attention, memory and information processing, with memory tests more heavily represented in the literature. Overall, the evidence in healthy young adults suggests that dietary supplementation with n-3 PUFA does not enhance cognitive function, with only a handful of studies showing clear significant benefits on memory [40], information processing [44] and attention [43]. Importantly, none of the above-mentioned studies adjusted for confounders, which is a strength of our study. There is evidence however that a threshold effect of low n-3 PUFA on cognition may explain non-significant findings [38, 46, 47]; hence the effects of n-3 PUFA supplementation in healthy participants may only be evident when cognitive performance is below average at baseline, or when n-3 PUFA status falls below a certain level (low or inadequate) [38]. Baseline or inadequate O3I levels has also been investigated in elderly cohorts, with O3I cut-off values suggested to define targets for future dementia trials. A study in dementia-free adults (70 years and over) reported an optimal cut-off of 5.3% for predicting notable cognitive decline and/or polyunsaturated fatty acid supplementation treatment response [48]. This cut-off is close to that for the lowest O3I tertile group in the current study (< 5.47%).

Several mechanisms of action have been proposed to explain the relationship between n-3 PUFA and cognitive function. First, PUFAs are known to facilitate effects on gene expression, especially in the central nervous system [1, 50–52]. One genetic factor that is attracting increasing attention is the Apolipoprotein E (APOE) genotype, which has been linked to Alzheimer’s disease. There is some evidence that this gene variant is also linked to cognitive performance in healthy young adults, but at present studies are conflicting and there is no consensus in the literature [49, 53, 54]. Secondly, it is known that n-3 PUFA has an important role in maintaining membrane integrity and fluidity, and neuronal functioning has been shown to be influenced by n-3 PUFA through a decrease in inflammatory pathways [1, 55, 56]. In support of the published reports, [57] C-reactive protein, an indicator of low-grade sustained inflammation, was lowest in women with the higher O3I in the present study, supporting a possible role of inflammation in determination of the attention domain of cognitive function.

Effects have also been found on dopaminergic neurotransmission, in particular, the mesocortical pathway has been implicated in attention, memory and executive function [56, 58, 59]. If these dopaminergic systems are altered via low n-3 PUFA, deficiency in this nutrient may contribute to reduced cognitive function. A recent study in diabetic rodents showed that administration of low doses of n-3 PUFAs could protect against neuronal damage in the hippocampus in type 2 diabetes and was associated with improved cognitive-behavioral performance and reduced inflammatory markers [55]. Furthermore, animal studies have reported evidence that n-3 PUFA (more specifically DHA) accumulates in areas of the brain involved in attention and memory, including the cerebral cortex and hippocampus [60, 61]. This observation is of interest, given that the current study found evidence of reduced cognitive function in the attention domain with lower O3I, albeit with no evidence of an effect on memory. There is limited information on the impact of short-term versus chronic inadequacy of n-3 PUFA or low O3I on cognition. However, in maternal and infant studies, there is evidence that there are crucial periods for adequate n-3 PUFA which may impact neurocognitive development [1, 3–5].

A major strength of this study is the recruitment of a large, healthy cohort, free of comorbidities. This study is one of the first to comprehensively exclude and/or adjust for a broad range of confounding variables when examining the influence of n-3 PUFA status on cognitive function in young women. Additionally, the use of erythrocyte n-3 PUFA (allowing for the calculation of the Omega-3 Index) provided an accurate and validated measure of longer-term n-3 PUFA status. Another major strength is the use of well-validated tools (IPAQ, IntegNeuro™) to assess outcome measures.

A limitation of this study is that it is not possible to deduce causal effects due to the cross-sectional study design. Limitations more generally in the examination of the effect of n-3 PUFA on cognitive function include use of plasma or serum n-3 PUFA to classify status. The long-term measure of erythrocyte n-3 PUFA may be more precise but is less accessible. A major limitation in the literature, particularly in the studies conducted in young adults, is that confounder adjustment for factors that may potentially influence cognition, including inflammation, obesity and physical activity, is rarely conducted. Previous systematic reviews assessing the effect of n-3 PUFA supplementation on cognitive function have also cited large heterogeneity in supplement interventions (the type of supplement, EPA/DHA content, duration of intervention, etc.) and variability in cognition assessment, as major limitations in this field [3, 10, 42]. There is also currently no consensus regarding the classification of cognitive tests and domains [16] and this lack may explain some of the discrepancies in results between studies.

## Conclusions

This study found reduced cognitive performance in the attention domain in young women with lower overall n-3 PUFA, although cognition scores were still within the normal range. Thus, the clinical significance of these findings warrants further investigation. Cognition testing pre- and post-intervention to rectify low n-3 PUFA status and assessment of genetic factors (particularly APOE4 and dopamine receptor genes) may help to further identify the relationship and mechanisms of action between n-3 PUFA status and cognitive performance.

## Data Availability

The datasets used and/or analyzed during the current study are available from the corresponding author on reasonable request.

## Abbreviations

ANCOVA: Analysis of covariance
ANOVA: Analysis of variance
APOE: Apolipoprotein E
BMI: Body mass index
CRP: C-reactive protein
DHA: Docosahexaenoic acid
EPA: Eicosapentaenoic acid
ES: Effect size
Hb: Hemoglobin
IPAQ: International Physical Activity Questionnaire
MET: Metabolic equivalent of the task
n-3 PUFA: Omega-3 polyunsaturated fatty acid
NATA: National Association of Testing Authorities
NW: Normal weight
O3I: Omega-3 Index
OB: Obese
PA: Physical activity
PUFA: Polyunsaturated fatty acid
SD: Standard deviation
SDT: Suggested dietary target
SE: Standard error
SF: Serum ferritin
α1GP: Alpha1-acid glycoprotein
